# The Impact of Changes in Depression on Cardiovascular Outcomes in Patients With Coronary Heart Disease

**DOI:** 10.1016/j.jacadv.2024.101348

**Published:** 2024-10-24

**Authors:** Nishant Vatsa, Josiah Bennett, Sonika Vatsa, Alireza Rahbar, Daniel A. Gold, Vardhmaan Jain, Matthew E. Gold, Alexander Razavi, Adithya Yadalam, Shivang Desai, Muhammad Owais, Joy D. Hartsfield, Yi-An Ko, Laurence Sperling, Viola Vaccarino, Puja K. Mehta, Arshed A. Quyyumi

**Affiliations:** a Emory Clinical Cardiovascular Research Institute, Division of Cardiology, Department of Medicine, Emory University School of Medicine, Atlanta, Georgia, USA; b J Willis Hurst Internal Medicine Residency Program, Department of Medicine, Emory University School of Medicine, Atlanta, Georgia, USA; c Rowan School of Osteopathic Medicine, Stratford, New Jersey, USA; d Department of Biostatistics and Bioinformatics, Rollins School of Public Health, Emory University, Atlanta, Georgia, USA; e Department of Epidemiology, Rollins School of Public Health, Emory University, Atlanta, Georgia, USA

**Keywords:** cardiovascular outcomes, coronary heart disease, depression

## Abstract

**Background:**

Depression is associated with major adverse cardiovascular events (MACE). Whether longitudinal changes in depression affect MACE in patients with coronary heart disease (CHD) remains unknown.

**Objectives:**

The authors evaluated the hypothesis that increasing or persistent depression predicts MACE in patients with CHD.

**Methods:**

At baseline, 3,483 Emory Cardiovascular Biobank participants (median age 65.5 years, 31.6% female) completed the Patient Health Questionnaire 8 (PHQ8) for depression evaluation. At 1 year, 2,639 of these event-free participants repeated the questionnaire. Depression was defined as a PHQ8 score >9 and change in depressive symptoms (Δ PHQ8) was year 1 score minus baseline PHQ8 scores. We categorized participants into never depression (both PHQ8 <10), new depression (baseline PHQ8 <10; 1-year PHQ8 >9), remitted depression (baseline PHQ8 >9; year 1 PHQ8 <10), and persistent depression (both PHQ8 >9) groups. Fine-Gray models with noncardiovascular death as the competing event and adjusted for demographics, CHD, and depression related factors evaluated how changes in depression affect MACE (cardiovascular death and MI).

**Results:**

Overall, the incidence of MACE was 14%, with 8.7% of those with follow-up PHQ8 having MACE. 2.9% had persistent depression, 4.5% had new depression, 10.8% had remitted depression, and 81.8% never had depression. Increasing depressive symptoms independently predicted MACE (Δ PHQ8 subdistribution HR: 1.06 [95% CI: 1.02-1.09], *P* < 0.001). Correspondingly, the incidence of MACE was higher in those with persistent (20.8%) or new depression (11.9%) than in those with remitted (9.4%) or never depression (8%) (*P* < 0.001). Compared to never depression, persistent depression independently predicted MACE (subdistribution HR: 2.78 [95% CI: 1.2-6.5], *P* = 0.017).

**Conclusions:**

Increasing or persistent depression predicts MACE in individuals with CHD.

Depression is one of the leading causes of morbidity worldwide and is estimated to impact 26% of patients with coronary heart disease (CHD).^1^^,^^2^ Depression is a risk factor for adverse cardiovascular (CV) outcomes in patients with recent cardiac events, the elderly, and those at risk for CHD.^3^, ^4^, ^5^, ^6^, ^7^, ^8^, ^9^, ^10^

Most studies have only linked depression measured at a single time point to major adverse cardiovascular events (MACE) and consequently fail to examine the impact of temporal changes in depressive symptoms on CV health.^3^, ^4^, ^5^, ^6^, ^7^, ^8^, ^9^, ^10^ Investigations examining longitudinal changes of depression on CV outcomes were done outside of the United States and included participants free of CHD. A Chinese study showed that the risk of CV disease in those with persistent depressive symptoms was 1.77 times the risk in those without any depressive symptoms, and a Norwegian population-based study showed that participants with persistently low depressive symptoms had 0.72 times the risk of mortality than those with persistently high depressive symptoms.^11^^,^^12^ Studies in the US have focused on the impact of changes in depression on stroke risk and have shown those with persistent depressive symptoms are at increased stroke risk compared to those with no depressive symptoms over time.^13^, ^14^, ^15^ However, the impact of persistent or worsening depressive symptoms on CHD outcomes in a high-risk US population with established CHD remains to be studied.^16^

Our study examined the longitudinal impact of changes in depression on major adverse CV outcomes in patients with CHD. We hypothesized that increasing or persistent depression increases the risk of MACE, defined as a composite of CV death and nonfatal MI, in this population.

## Methods

### Study population

We leveraged data from the Emory Cardiovascular Biobank, a registry of participants aged between 20 and 90 years old who underwent clinically indicated catheterization at Emory-affiliated sites between 2003 and 2022. Participants received catheterization for various reasons, including heart failure evaluation, workup for ACS, angina, preoperative evaluation, abnormal stress tests, and transplant evaluation or follow-up.^17^ We excluded patients with organ transplantation, acute MI on enrollment, normal coronary angiograms by visual assessment from blinded operators, and those without baseline depression assessments or lack of CV outcome data.

**Figure 1:**
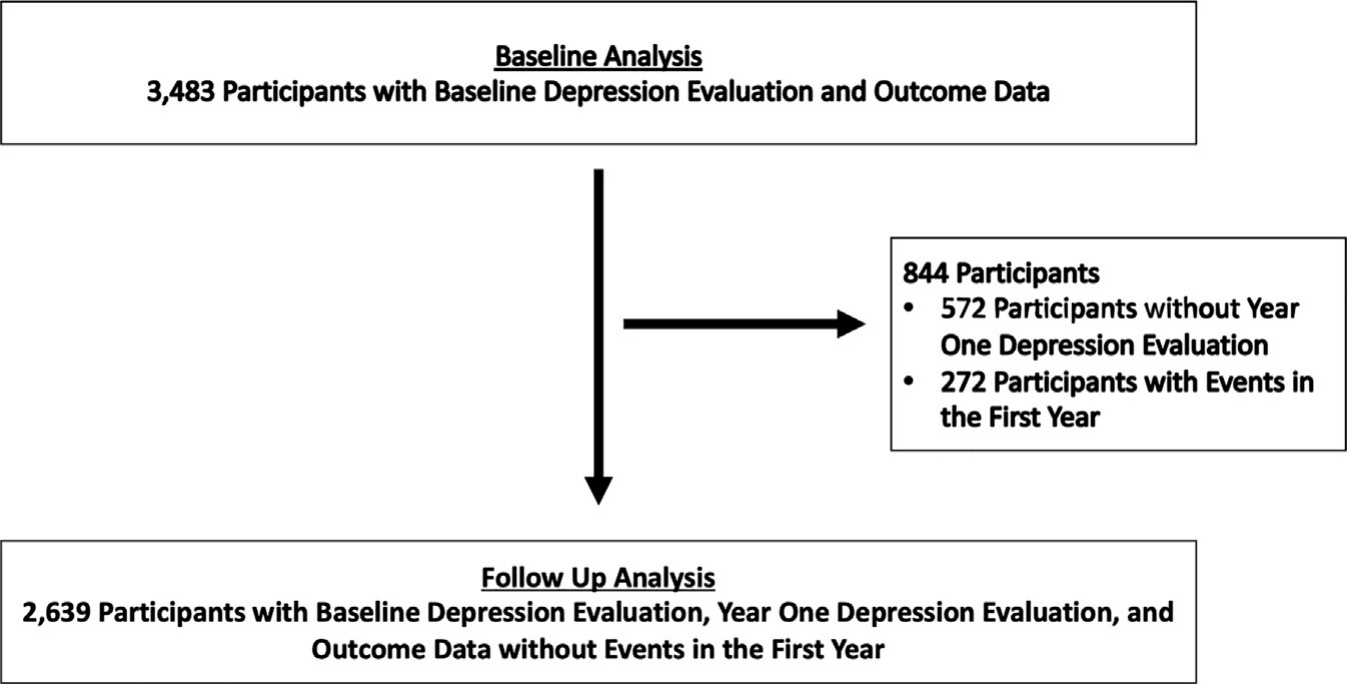
**Participant Selection** Participant selection for analyses evaluating the impact of baseline, year-1, and change in depressive symptoms or status on CV death or MI. We excluded individuals with baseline depression assessments who did not complete follow-up depression assessments or had CV death or MI before follow-up from analyses evaluating the impact of year 1 and change depression or depressive symptoms on CV death or MI.

Consequently, 3,483 participants were included in the analyses examining the association between baseline depression and CV death or MI. In analyses investigating how depression at 1-year follow-up and the changes in depression affected future CV death or MI, we excluded those without depression assessments at 1-year follow-up and those with events before their 1-year follow-up (Figure 1). The Institutional Review Board at Emory University approved this study, and all participants signed informed consent.

### Depression assessment

To measure depression, we used the Patient Health Questionaire-8 (PHQ8), an 8-item validated self-administered questionnaire that assesses depressive symptoms and aids in diagnosing major depression.^18^, ^19^, ^20^ The questionnaire consists of 8 items asking about the frequency of depression-associated symptoms and omits the ninth item from PHQ9, asking about suicidality. Based on responses, each item is scored from 0 (“not at all”) to 3 (“nearly every day”), and scores are aggregated to generate a total score, which ranges from 0 to 24. Compared to expert evaluations, PHQ8 scores above 9—a cutoff ubiquitously used in clinical and research settings—have above 85% sensitivity and specificity in diagnosing depression.^18^, ^19^, ^20^, ^21^, ^22^ Participants completed the PHQ8 in person upon enrollment and over the phone at 1-year follow-up. We used PHQ8 scores at baseline and 1-year follow-up to approximate depressive symptoms at those time points. We calculated the change in depressive symptoms as the difference between 1-year and baseline PHQ8 scores (Δ PHQ8). We labeled those with a PHQ8 score of greater than 9 as having depression status. We further categorized individuals with PHQ8 ≤9 at both time points as those with “never depression,” baseline PHQ8 >9 and 1-year PHQ8 ≤9 as those with “remitted depression,” baseline PHQ8 ≤9 and 1-year PHQ8 >9 as those with “new depression,” and PHQ8 >9 at both time points as individuals with “persistent depression.” Upon enrollment and at the 1-year follow-up, we gathered information on depression therapies by asking participants if they were receiving counseling or were taking antidepressants.

### Outcomes and follow-up

Emory Cardiovascular Biobank participants were followed for 5 years after baseline depression assessments and for 4 years after 1-year depression assessments for incident CV death or nonfatal MI. We conducted follow-ups for CV outcomes through phone contact, medical record review, state records, and the Social Security Death Index.^17^ Medical record review and communication with the participant's family were utilized to ascertain the cause of death. CV death was adjudicated by a cardiologist blinded to other study data.^17^ CV death was defined as ischemic CV-related mortality, which includes fatal stroke, MI, or sudden death due to unknown or presumed cardiac etiology in at-risk patients.^17^ The fourth universal definition of MI was used to define MI, which required participants to have clinical evidence of acute myocardial injury (symptoms, acute ischemic electrocardiogram changes, ischemic imaging findings, or evidence of coronary thrombus through angiography/autopsy) and a rise and fall of troponin levels with 1 value above the 99th percentile value.^23^

### Covariates

Covariates collected and included in modeling were variables associated with CV disease or depression. Relevant medical, demographic, CVD risk factor history, and medication use information were obtained by participant interviews and confirmed with medical records review upon enrollment. Anthropometric measures and serum hsCRP serum levels were also collected at enrollment through physical exams and blood draws. Kidney function was determined by serum creatinine measurements at or before enrollment. Covariates with over 10% missing data were excluded from modeling. Model covariates with 5% to 10% missing values were imputed via the multiple imputations by chained equations (mice) R package.^24^ As a result, body mass index (BMI) (5.2% missing) and depression history before enrollment (8.1% missing) were the only model covariates imputed, while hsCRP (13.6% missing) was excluded from modeling.

### Statistical analysis

Baseline characteristics of our sample are presented as proportions for categorical variables. Normality of continuous variables were assessed with the Shapiro-Wilk test. Normal continuous variables are presented as mean ± SD and non-normal continuous variables are presented as median (IQR). As appropriate, comparisons between groups were performed with the chi-square test, *t*-test, or Mann-Whitney U test.

We used multiple Fine and Gray subdistribution hazard models with non-CV disease death as a competing event to assess the effect of depressive symptoms at both time points (baseline and 1-year PHQ8) and changes in depressive symptoms (Δ PHQ8) on CV death or MI. Model 1 included baseline depressive symptoms, 1-year depressive symptoms, or Δ PHQ8. Model 2 included covariates from model 1 and demographics, such as age, sex, and race. Model 3 included covariates from model 2 and CHD-relevant factors, such as BMI, history of hyperlipidemia, history of hypertension, history of diabetes, history of heart failure, history of smoking, glomerular filtration rate, CAD severity as measured by the number of vessels with obstructive (>50% stenosis) disease, aspirin use, and statin use. Model 4 included covariates from Model 3 and other depression-relevant indicators, such as self-reported counseling or medication for depression and a history of depression before enrollment. We used model 4 to assess the impact of baseline, 1 year, and change in depression status on incident MACE. Models assessing 1-year depressive symptoms or status and changes in depressive symptoms or status were also adjusted for baseline depressive symptoms. Based on the Schoenfeld residual test, the above models met the proportional hazards assumptions.

We utilized Cox proportional hazards models with covariates from model 4 for interaction analyses, evaluating whether demographics, CV risk factors, depression therapy, history of depression before enrollment, baseline depression status, or CAD severity modified the effect of Δ PHQ8 on CV death or MI. Subgroup analyses were performed on interactions with *P* values less than 0.1. We completed all analyses using SPSS 28.0.1.0 and R 4.2.3 (R Foundation for Statistical Computing).

## Results

### Sample characteristics

The 3,483 participants with baseline depression data and complete follow-up for CHD outcomes had a median age of 65.5 (IQR: 57.3-73.1) years, were predominantly male (68.4%), White (78.5%), had a high prevalence of depression (n = 518, 14.9%) with a median PHQ8 of 3,^1^^,^^7^ and had a median survival time of 1,826 [(IQR: 777-1,826) days. Of baseline participants, 487 (14%) experienced incident CV death or MI. Those with baseline depression were younger, more likely to be female, to have diabetes, a history of smoking and heart failure, and had higher BMI.

Of the 3,483 patients with baseline depression data, 2,639 individuals who did not have an adverse event during the first year completed a follow-up PHQ8 at the 1-year follow-up. The follow-up cohort had a median age of 65.8 (IQR: 58.4-72.9) years, was mostly male (68%), and had low baseline and 1-year PHQ8 scores (3 [IQR: 0-6] and 2 [IQR: 0-5], respectively), and 230 (8.7%) had incident CV death or MI. Among the follow-up cohort, 77 (2.9%) had persistent depression (baseline and 1-year PHQ8 >9), 118 (4.5%) had new onset of depression (baseline PHQ8 ≤9, 1-year PHQ8 >9), 286 (10.8%) had remitted depression (baseline PHQ8 >9, 1-year PHQ8 ≤9), and 2,158 (81.8%) had never depression (baseline and 1-year PHQ8 ≤9) at 1-year follow up. Those with new or persistent depression were younger, more likely to be female, and more likely to have diabetes and heart failure than those who never had depression. Those with persistent depression had the highest hsCRP levels and were the least likely to have severe coronary artery stenosis (≥70% stenosis) (Table 1).

**Table 1:** Sample Characteristics

	Overall (N = 2,639)	Depression Status Change				*P* Value^a^
		Never Depression (n = 2,158)	Remitted Depression (n = 286)	New Depression (n = 118)	Persistent Depression (n = 77)	
Baseline demographics						
Age (y)	65.8 (58.4-72.9)	66.2 (59.1-73.1)	64.4 (55.9-72.5)	64.6 (59.2-72)	59.4 (52.3-68.3)	<0.001
Sex						<0.001
Female	846/2,638 (32%)	645/2,157 (30%)	114/286 (40%)	51/118 (43%)	36/77 (47%)	
Male	1,792/2,638 (68%)	1,512/2,157 (70%)	172/286 (60%)	67/118 (57%)	41/77 (53%)	
Race						0.33
White	2,121/2,638 (80%)	1747/2,157 (81%)	219/286 (77%)	95/118 (81%)	60/77 (78%)	
Black	428/2,638 (16%)	336/2,157 (16%)	55/286 (19%)	20/118 (17%)	17/77 (22%)	
Hispanic	15/2,638 (0.6%)	12/2,157 (0.6%)	1/286 (0.3%)	2/118 (1.7%)	0/77 (0%)	
Asian	42/2,638 (1.6%)	37/2,157 (1.7%)	4/286 (1.4%)	1/118 (0.8%)	0/77 (0%)	
Native American	2/2,638 (0.1%)	2/2,157 (0.1%)	0/286 (0%)	0/118 (0%)	0/77 (0%)	
Other	30/2,638 (1.1%)	23/2,157 (1.1%)	7/286 (2.4%)	0/118 (0%)	0/77 (0%)	
Baseline cardiovascular risk factors						
Diabetes (%)	958/2,623 (37%)	749/2,148 (35%)	121/284 (43%)	54/116 (47%)	34/75 (45%)	0.002
Hypertension (%)	2,107/2,627 (80%)	1,717/2,150 (80%)	234/285 (82%)	93/117 (79%)	63/75 (84%)	0.68
Hyperlipidemia (%)	2,039/2,626 (78%)	1,663/2,150 (77%)	232/284 (82%)	87/117 (74%)	57/75 (76%)	0.30
Heart failure (%)	716/2,639 (27%)	551/2,158 (26%)	92/286 (32%)	42/118 (36%)	31/77 (40%)	<0.001
Smoking (%)	1,792/2,639 (68%)	1,447/2,158 (67%)	200/286 (70%)	83/118 (70%)	62/77 (81%)	0.066
GFR (mL/min)	73.9 [59.4, 88.2]	74.2 [59.9, 87.9]	73 [59.2, 88.5]	72 [53.6, 88.6]	73.3 [60.7, 94.9]	0.70
BMI (kg/m^2^)	28.9 [25.7, 33]	28.7 [25.6, 32.4]	30.6 [26.5, 34.6]	30.5 [27, 34.9]	28.9 [25, 35.9]	<0.001
hsCRP (mg/dL)	2.4 [1, 5.8]	2.2 [0.9, 5.3]	3.5 [1.5, 8.1]	3.5 [1.4, 6.2]	4.9 [1.7, 8.9]	<0.001
Number with 30% stenosis	2,382/2,533 (94%)	1,951/2,068 (94%)	254/276 (92%)	107/114 (94%)	70/75 (93%)	0.45
Number with 50% stenosis	2,094/2,413 (87%)	1,722/1,973 (87%)	229/268 (85%)	86/104 (83%)	57/68 (84%)	0.41
Number with 70% stenosis	1,833/2,317 (79%)	1,518/1,896 (80%)	195/258 (76%)	78/101 (77%)	42/62 (68%)	0.045
Baseline medications						
Statin	2,114/2,639 (80%)	1,735/2,158 (80%)	224/286 (78%)	94/118 (80%)	61/77 (79%)	0.86
Aspirin	2,188/2,639 (83%)	1,793/2,158 (83%)	231/286 (81%)	97/118 (82%)	67/77 (87%)	0.59
Depression history						
Baseline PHQ8 Score	3 [0, 6]	2 [0, 4.6]	13 [11, 15.8]	4.0 [2, 6]	15 [11, 18]	<0.001
Year 1 PHQ8 Score	2 [0, 5]	2 [0, 4]	3.5 [1, 6]	12 [10, 14]	12 [10, 15]	<0.001
History of depression	429/2,426 (18%)	257/1,973 (13%)	104/270 (39%)	29/109 (27%)	39/74 (53%)	<0.001
Depression treatment or counseling	488/2,632 (19%)	314/2,152 (15%)	94/285 (33%)	44/118 (37%)	36/77 (47%)	<0.001
Outcomes						
CVD death	141/2,639 (5.3%)	102/2,158 (4.7%)	17/286 (5.9%)	11/118 (9.3%)	11/77 (14%)	0.002
MI	122/2,639 (4.6%)	95/2,158 (4.4%)	16/286 (5.6%)	6/118 (5.1%)	5/77 (6.5%)	0.56

Values are median (IQR) or n/N (%). *P* value compared those with never, remitted, new, and persistent depression. Significant *P* < 0.05.

a Kruskal–Wallis rank sum test, Pearson’s chi-square test, Fisher’s exact test.

### Relationship between baseline, 1 year, and changes in depression (PHQ8 >9) and outcomes

**Figure 2:**
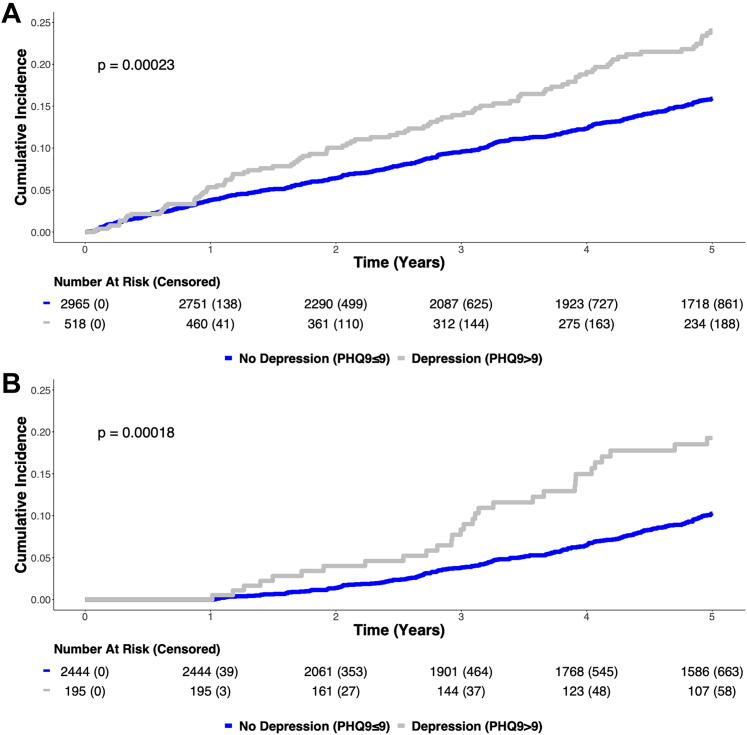
MACE Cumulative Incidence Curves by Depression Status Kaplan–Meier unadjusted cumulative incidence curves of participants with depression (PHQ8 >9, grey) and no depression (PHQ8 ≤9, blue) at enrollment (A) and year 1 follow-up (B). Significant differences are denoted by log-rank *P* < 0.05. PHQ8 = Patient Health Questionnaire 8.

The cumulative incidence of CV death or MI was higher in those with baseline depression (baseline PHQ8 >9) than those without baseline depression (log-rank *P* < 0.001), wherein 96 individuals (18.5%) with baseline depression had CV death or MI compared to 391 individuals (13.2%) without baseline depression experiencing subsequent CV death or MI (Figure 2A). Similarly, those with 1-year depression (1-year PHQ8 > 9) had a higher incidence of CV death or MI than those without 1-year depression (log-rank *P* < 0.001), as 30 participants (15.4%) with 1-year depression had incident CV death or MI after follow-up as opposed to 200 individuals (8.2%) without 1-year depression that had CV death or MI after follow-up (Figure 2B). Consequently, the incidence of CV death or MI was higher among those with persistent (n = 16, 20.8%) or new depression (n = 14, 11.9%) versus those with remitted (27 individuals, 9.4%) or never (n = 173, 8%) depression (log-rank *P* < 0.001) (Figure 2C).

The above differences were driven by the high incidence of CV death among those with baseline or 1-year depression and among those with persistent or new depression. Those with depression had a significantly higher incidence of CV death compared to those without depression at baseline (n = 68 (13.1%) versus 270 individuals (9.1%), *P* = 0.006) and 1-year (n = 22 (11.3%) versus 119 individuals (4.9%), *P* < 0.001). Correspondingly, the incidence of CV death among those with persistent (n = 11, 14.3%) or new (n = 11, 9.3%) depression was significantly higher than those with remitted (n = 17, 5.9%) or never (n = 102, 4.7%) depression (*P* = 0.001) (Table 1).

After full adjustment for demographics, CV risk factors, medications, and depression risk factors, the results were similar. Baseline (adjusted subdistribution HR (sHR): 1.41 [95% CI: 1.11-1.79], *P* = 0.005) and 1-year (adjusted sHR [95% CI]: 1.91 [95% CI: 1.25-2.91], *P* = 0.003) depression independently predicted incident CV death or MI in the subsequent years. Additionally, the hazard of CV death or MI among those with persistent depression was 2.78 times the hazard of CV death or MI among those with never depression (adjusted sHR: 2.78 [95% CI: 1.2-6.5], *P* = 0.017).

### Relationship between baseline, 1 year, and changes in depressive symptoms (ΔPHQ8) and outcomes

**Figure 3:**
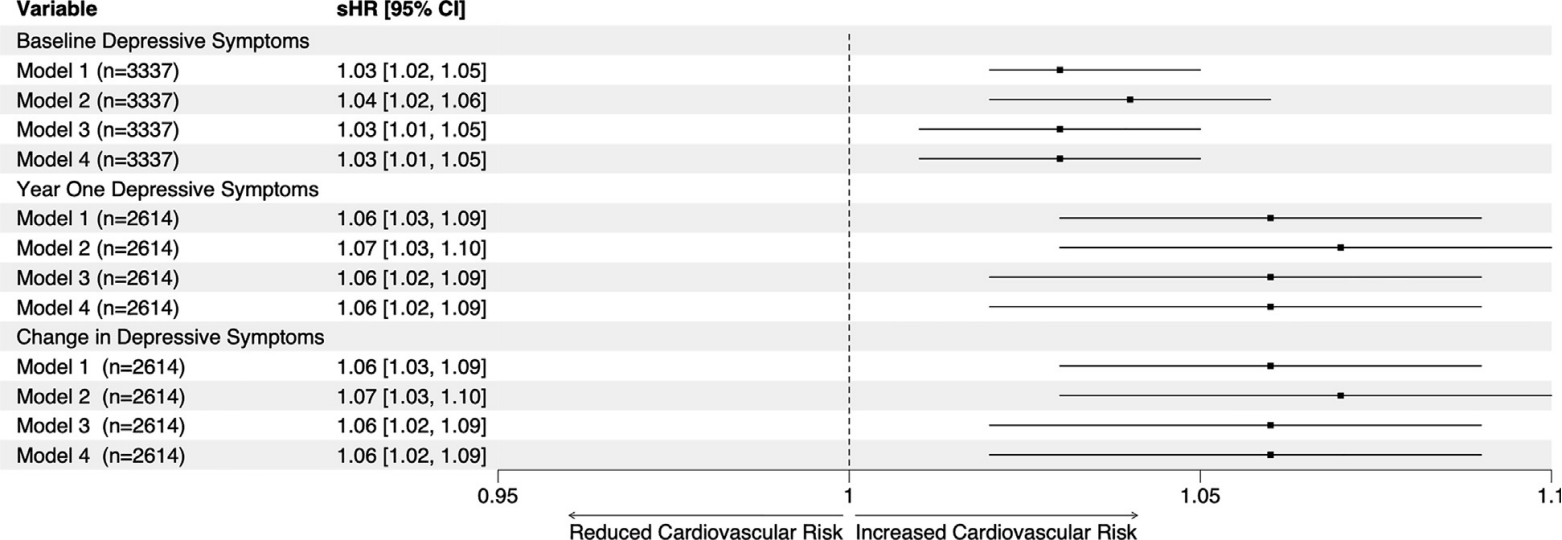
**sHR of Depressive Symptoms on MACE** Ratios for baseline, year 1, and change in depressive symptoms (Δ PHQ8) were assessed by Fine and Gray models with the composite of CV death or MI as the event of interest and non-CV death as the competing event. Model 1 is the univariate model, model 2 is adjusted for demographics, model 3 is adjusted for model 2 covariates and cardiovascular risk factors, and model 4 is adjusted for model 3 covariates and depression risk factors. PHQ8 = Patient Health Questionnaire 8; sHR = subdistribution HR.

**Figure 4:**
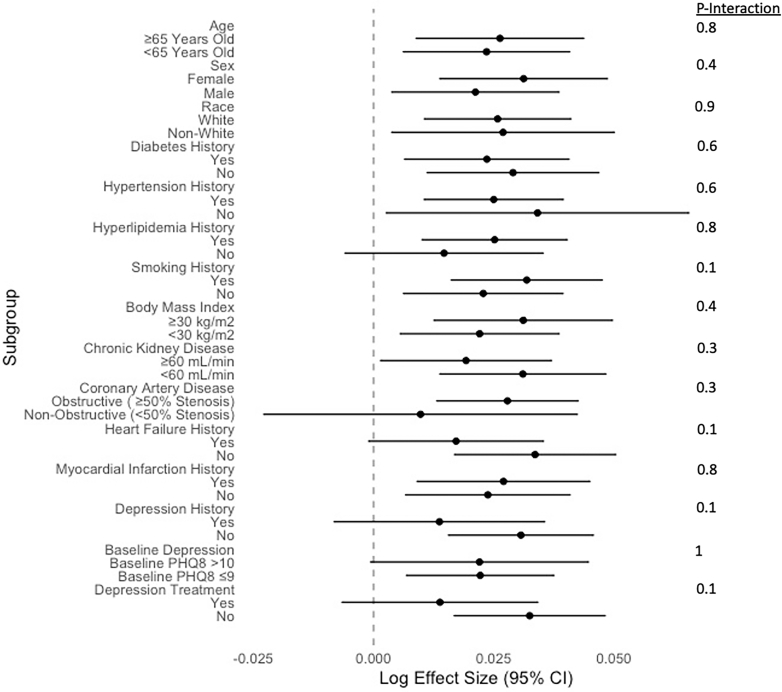
**Interaction Between ΔPHQ8 and Covariates on MACE** Pooled interaction effects between change in depressive symptoms (ΔPHQ8) and listed covariates on CV death or MI. Pooled forest plots showing adjusted log effect sizes of change in depressive symptoms (ΔPHQ8) on CV death or MI by subgroup strata, which were calculated by Cox proportional hazards models adjusted for demographics, cardiovascular risk factors, and depression risk factors. Significant interactions have *P* for interaction<0.05. PHQ8 = Patient Health Questionnaire 8.

Baseline and 1-year depressive symptoms (baseline and 1-year PHQ8) were independently predictive of CV death or MI in models adjusted for demographics, CV risk factors, medications, and depression risk factors. At baseline, for every 5-point increase in the PHQ8 score, there was a 15% increase in the hazard of CV death or MI (adjusted sHR, 1-point increase: 1.03 [95% CI: 1.01-1.05], *P* = 0.009) (Figure 3). For every 5-point increase in 1-year PHQ8 score, there was a 30% increase in the hazard of CV death or MI (adjusted sHR, 1-point increase: 1.06 [95% CI: 1.02-1.09], *P* = 0.0008) (Figure 3). As a result, 1-year changes in depressive symptoms (Δ PHQ8) were also predictive of CV death or MI. Those with worsening depressive symptoms (increasing ΔPHQ8) over 1 year were at increased risk of CV death or MI independent of CV risk factors, demographics, medication use, and depression risk factors (adjusted sHR, 1-point increase: 1.06 [95% CI: 1.02-1.09], *P* = 0.0008). The increased risk of CV death or MI among those with worsening depressive symptoms was consistent across demographics, CV disease risk factors, CAD severity, depression treatment, and prior depression status subgroups (Figure 4).

## Discussion

In this cohort with CHD, we demonstrate a time–dose effect of depression and depressive symptoms on MACE. Depression independently predicted CV death or MI across multiple time points such that increasing or persistent depression over time independently increased the risk of MACE. Likewise, more severe depressive symptoms at different time points predicted MACE, so those with worsening depressive symptoms over time were at increased risk of MACE. The relationship between longitudinal changes in depressive symptoms and MACE was independent of and not modified by CHD risk factor characteristics, demographics, depression therapy, depression history, medications, or CAD severity. These data demonstrate the prognostic utility of repeated PHQ8 screenings to risk-stratify patients with CHD.

Our study expands findings from previous investigations, which have associated single-time assessments of depression with MACE in patients with low to high CVD risk profiles, by establishing the importance of longitudinal changes in depression on CV health in patients with CHD.^3^, ^4^, ^5^, ^6^, ^7^, ^8^, ^9^, ^10^ Our study findings are consistent with population studies in healthy individuals and therapeutic or observational studies in post-MI individuals that showed a higher risk of CV events among those with persistent depression.^11^, ^12^, ^13^, ^14^, ^15^^,^^25^^,^^26^ We uniquely show a similar association between worsening or persistent depression and CV outcomes in patients with stable CHD, who have a higher risk than healthy populations but reduced cardiac risk to post-ACS cohorts.

In our cohort, the detrimental impact of worsening or persistent depression on CV health was independent of CV risk factors. Hence, mechanisms outside the clinical risk factors included in our models could facilitate trends in our study. Those with persistent depression have significantly higher hsCRP concentrations than those who never had depression, so depression-induced inflammation could be contributing to our findings. Vaccarino and Kop have shown that inflammation partially accounts for the association between depression and CV disease.^27^^,^^28^ Therefore, the elevated risk of MACE in those with persistent or worsening depression could be mediated by prolonged and intensified inflammation, which provides the substrate for MACE through heightened platelet activity and endothelial dysfunction.^29^, ^30^, ^31^ The altered hypothalamus–pituitary–adrenal axis and increased sympathetic activity seen in animal and human models of depression could also be contributing to the harmful impact of persistent or worsening depression on CV risk.^28^^,^^32^^,^^33^ Future studies investigating how changes in sympathetic drive and inflammation mediate the longitudinal effect of depressive symptoms on CV events would be illuminating.

Post-MI patients with depression have reduced compliance with low-fat diets, regular exercise, and medication adherence.^34^^,^^35^ So, adverse behavioral and lifestyle practices among those with persistent or increasing depression in this cohort could partially explain adverse CV outcomes in this group. Rieckmann et al. revealed subsequent improvements in medication adherence with decreasing depressive symptoms.^35^ These results and previous literature contextualize the benefit of serial depression screening and emphasize the need for these screenings in preventive cardiac care—sentiments previously expressed by the American Heart Association.^36^^,^^37^ Regular screening could lead to timely recognition and intervention of increasing or persistent depression among those with CHD, thereby mitigating risky lifestyle practices and promoting healthy behaviors that reduce MACE in these individuals.^34^^,^^35^

The impact of non–lifestyle therapies that mitigate worsening or persistent depression on CV health needs further clarification. Cognitive behavioral therapy has been shown to decrease depression in patients and improve HDL levels in patients with CHD.^38^ Trials examining antidepressant medications’ impact on CV outcomes in post-ACS patients have produced mixed results.^3^, ^4^, ^5^, ^6^, ^7^, ^8^, ^9^, ^10^, ^11^, ^12^, ^13^, ^14^, ^15^^,^^25^^,^^39^, ^40^, ^41^ Sweda and colleagues’ meta-analysis failed to show mortality or MI benefit of antidepressant therapy in post-ACS patients; however, it included trials, such as MIND-IT and SADHART, with treatment-resistant depression and studies with patients without depression—rendering the results controversial.^25^^,^^39^, ^40^, ^41^ When restricted to trials that included post-ACS patients with concomitant depression, those treated with antidepressants had less incidence of recurrent MI than those without therapy.^41^ Furthermore, in recent trials and secondary analyses, antidepressants reduced MACE in post-MI patients with treatment-responsive depression compared to their counterparts with treatment-refractory depression or placebo.^42^, ^43^, ^44^, ^45^ Thus, there may be cardiac benefit of antidepressants and cognitive behavioral therapy in select at-risk individuals with persistent depression, similar to our cohort. More clinical trials that elucidate the impact of these therapies on CV health in those with CHD are warranted.

### Study Limitations

Although the American Heart Association recommends items from the PHQ8 in depression screening, our use of a self-reported questionnaire without evaluations from mental health experts could have led to misclassification and recall bias.^36^ Our study had 1 follow-up depression and no follow-up clinical risk factor assessment after baseline evaluations, so we cannot ascertain how depression changes after our follow-up assessment or changes in clinical risk factors impacted our results. Consequently, future studies investigating longitudinal changes in depression's effect on CV outcomes with longer follow-up and closer depression, clinical, and socioeconomic risk factor surveillance are needed. A strength of our study was our analysis included a large sample of individuals with baseline depression assessments, follow-up depression assessments, and outcome data. However, 572 individuals with baseline depression assessments did not complete follow-up assessments, so there could have been selection bias, as these individuals may represent a group with increased barriers to health care. Lastly, our study includes participants from the Atlanta metropolitan area and thus may not be generalizable to other geographic populations.

## Conclusions

Longitudinal changes in depression impact CV outcomes in patients with CHD, where those with persistent or worsening depression are at increased risk of MACE, independent of other factors. Persistent or worsening depression may contribute to residual risk in patients with CHD. Serial screenings of depressive symptoms, irrespective of a previous history of depression or depression management, could help to risk stratify patients with CHD (Central Illustration).

Perspectives **COMPETENCY IN MEDICAL KNOWLEDGE:** Persistent or worsening depression independently predicts MACE in an American cohort with coronary heart disease across demographic and clinical risk factor profiles. These results emphasize the need for serial depression screenings of those with coronary artery disease in preventive clinical care, which could lead to timely recognition and intervention of persistent or worsening depression that could mitigate residual cardiac risk in this group.

**TRANSLATIONAL OUTLOOK:** Future prospective studies that examine how depression medication and psychotherapy impact cardiovascular health in those with coronary artery disease would further illuminate the effect of longitudinal changes in depression on cardiovascular outcomes in this group.

**Central Illustration:**
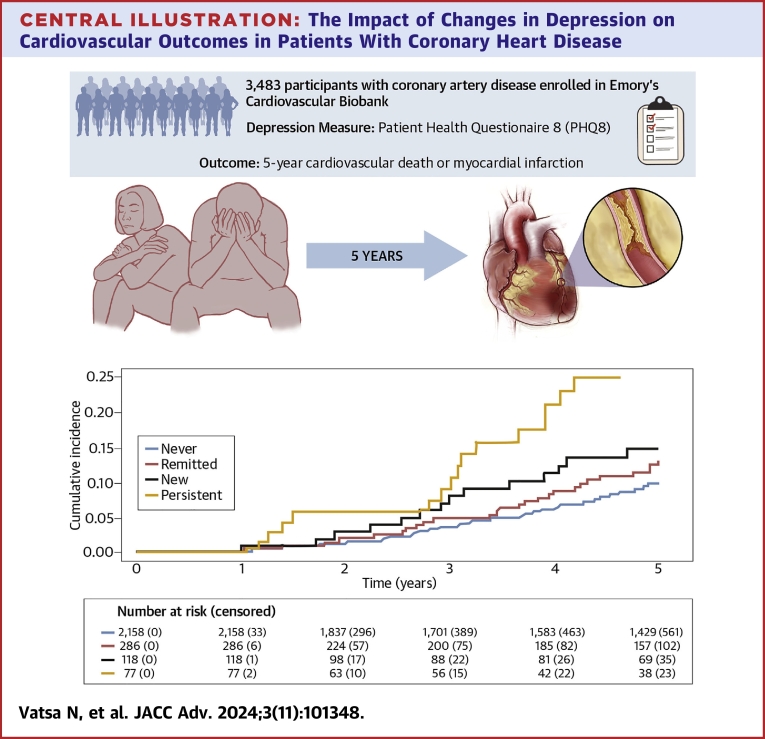
**The Impact of Changes in Depression on Cardiovascular Outcomes in Patients With Coronary Heart Disease** Persistent depression is associated with MACE. Kaplan–Meier unadjusted cumulative incidence curves show a significantly higher incidence of MACE among those with persistent depression (gold) or new depression (black) versus those with remitted depression (red) or never depression (blue).

## Funding support and author disclosures

Dr Vatsa is supported by the 10.13039/100013726Abraham J. & Phyllis Katz Foundation (Atlanta, GA), 10.13039/100000002NIH–10.13039/100000050NHLBI grant 1R01HL157311, and T32 HL130025. Dr Vaccarino is supported by R01 HL109413 and R01 HL163998. All other authors have reported that they have no relationships relevant to the contents of this paper to disclose.
